# p38 MAPK Inhibitor Insufficiently Attenuates HSC Senescence Administered Long-Term after 6 Gy Total Body Irradiation in Mice

**DOI:** 10.3390/ijms17060905

**Published:** 2016-06-08

**Authors:** Lu Lu, Yue-Ying Wang, Jun-Ling Zhang, De-Guan Li, Ai-Min Meng

**Affiliations:** 1 Institute of Radiation Medicine, Chinese Academy of Medical Science and Peking Union Medical Collage, Tianjin Key Laboratory of Radiation Medicine and Molecular Nuclear Medicine, Tianjin 300192, China; lulu@irm-cams.ac.cn (L.L.); wangyueying2010@163.com (Y.-Y.W.); zhangjunling@irm-cams.ac.cn (J.-L.Z.); 2 Institute of Laboratory Animal Science, Chinese Academy of Medical Science and Peking Union Medical Collage, Beijing 100021, China

**Keywords:** p38, ionizing radiation, bone marrow, long-term myelosuppression

## Abstract

Senescent hematopoietic stem cells (HSCs) accumulate with age and exposure to stress, such as total-body irradiation (TBI), which may cause long-term myelosuppression in the clinic. However, the methods available for long-term myelosuppression remain limited. Previous studies have demonstrated that sustained p38 mitogen-activated protein kinases (p38 MAPK) activation in HSCs following exposure to TBI in mice and the administration of its inhibitor twenty-four hours after TBI may partially prevent long-term myelosuppression. However, long-term myelosuppression is latent and identified long after the administration of radiation. In this study, we investigated the effects of SB203580 (a small molecule inhibitor of p38 MAPK) on long-term myelosuppression induced by TBI. Mice with hematopoietic injury were injected intraperitoneally with SB203580 every other day five times beginning 70 days after 6 Gy of ^137^Cs γ ray TBI. Our results at 80 days demonstrated that SB203580 did not significantly improve the TBI-induced long-term reduction of peripheral blood cell and bone marrow nucleated cell (BMNC) counts, or defects in hematopoietic progenitor cells (HPCs) and HSC clonogenic function. SB203580 reduced reactive oxygen species (ROS) production and p-p38 expression; however, SB203580 had no effect on p16 expression in the HSCs of mice. In conclusion, these findings suggest that treatment with SB203580 70 days after TBI in mice inhibits the ROS-p38 oxidative stress pathway; however, it has no therapeutic effect on long-term myelosuppression induced by TBI.

## 1. Introduction

Senescent cells accumulate with aging and multiple physiological and pathological processes. Cellular senescence is multifunctional, including protection against cancer and participation in complex biological processes, such as embryonic development [1], tissue repair [2], aging and age-related disorders [3]. Therefore, effective therapeutic strategies for the treatment of age-related diseases and improvements in healthy lifespans are needed.

Cell senescence primarily occurs via two signaling pathways, including the p53-p21 pathway, which is activated by DNA damage or shortened telomeres, and the p16-Rb pathway, which is activated by the p38 MAPK cascade. The activation of these pathways may induce senescence, and the p38-p16 pathway plays an important role in the senescence pathway [4,5]. Our previous study demonstrated that hematopoietic stem cells (HSCs) undergo senescence *in vitro* and *in vivo* following exposure to ionizing radiation (IR) [6,7]. The senescent HSCs induced by IR expressed increased senescence-associated-β-galactosidase (SA-β-gal) activity and p16 levels. In addition, the p38 expression and ROS levels increased [8].

Increasing evidence indicates that the p38-p16 pathway plays an important role in the regulation of HSC self-renewal and the remission of hematopoietic cell senescence induced by IR *in vitro* and *in vivo* [8,9]. The inhibition of p38 activation with the small molecule inhibitor SB203580 promotes *ex vivo* HSC expansion [10]. Moreover, the inhibition of p38 MAPK with SB203580 24 h after TBI attenuates IR-induced residual BM damage [9]. Furthermore, chemical inhibition of p38 rejuvenates aged satellite cells and promotes muscle regeneration following injury [11]. In contrast to acute myelosuppression, long-term myelosuppression is latent [7,12], is long-lasting, and exhibits little tendency for recovery [13]. Therefore, we investigated whether administration of the p38 inhibitor SB203580 after ionizing radiation-induced HSC senescence may ameliorate TBI-induced long-term myelosuppression.

## 2. Results

### 2.1. Effects of SB203580 on Peripheral Blood Cells after TBI

**Figure 1:**
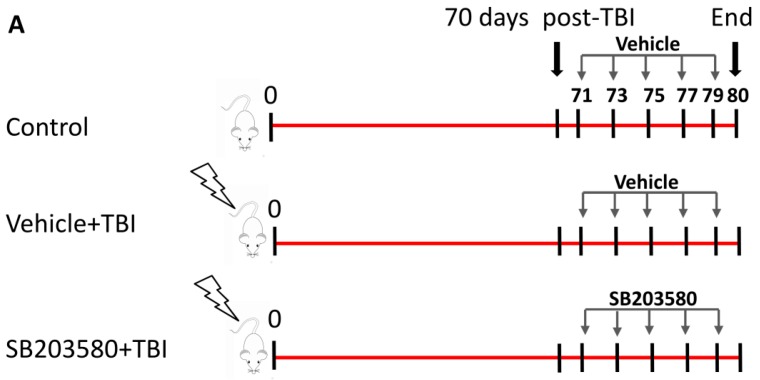

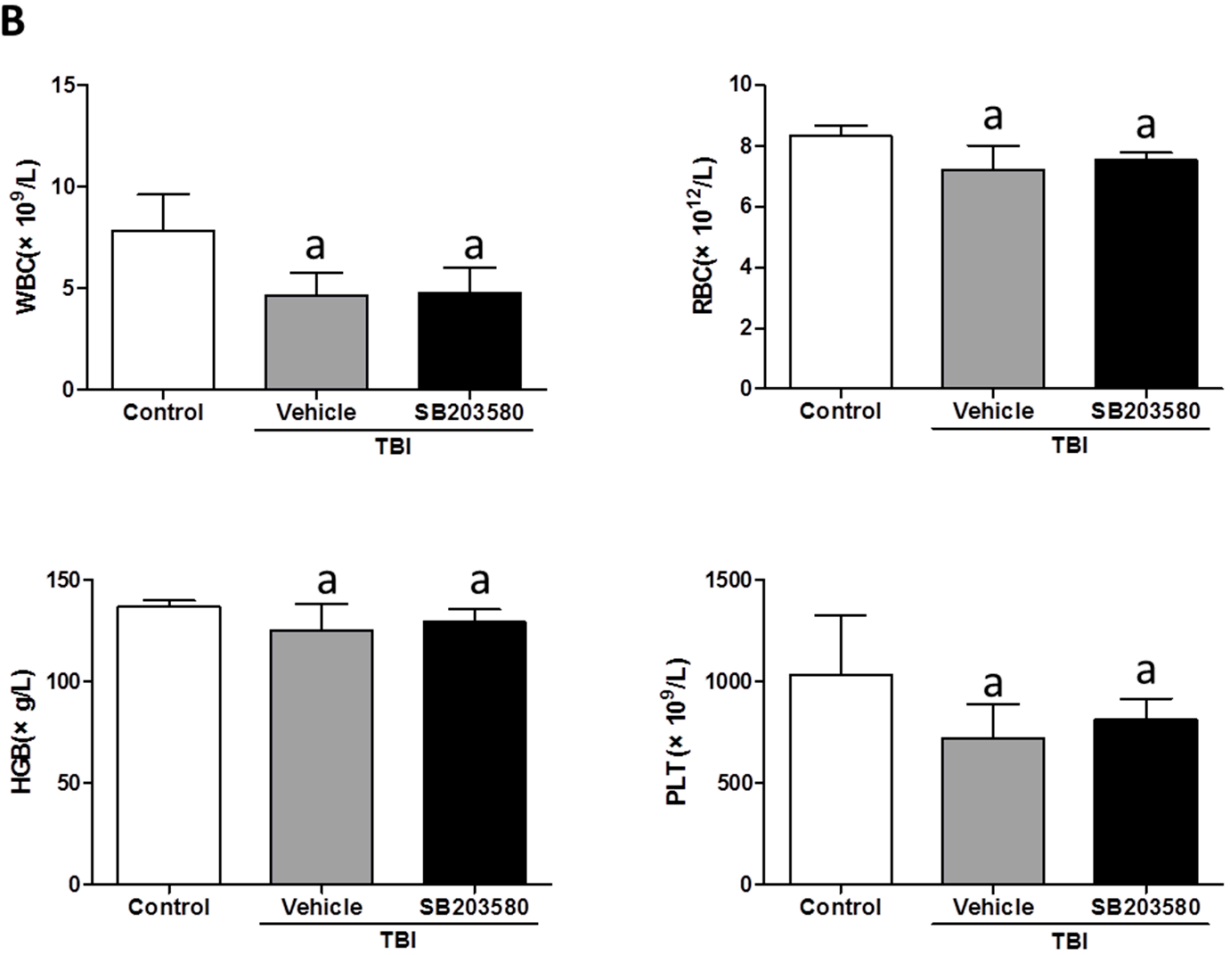
Effects of SB203580 on TBI-induced myelosuppression. (**A**) Mice were sham-irradiated or irradiated with 6 Gy TBI; 70 days after TBI, they were treated with SB203580 or vehicle every other day for five times as shown. Control mice were sham-irradiated and received injections of vehicle; (**B**) Peripheral blood cells were counted 80 days after 6 Gy TBI. The number of white blood cells (WBCs), red blood cells (RBCs), hemoglobin (HGB), and platelets (PLT) in the peripheral blood were quantified 80 days after 6 Gy TBI. The data are presented as the means ± SD. *n* = 12 mice/group. ^a^ *p* < 0.05 *vs.* control.

Previous studies have demonstrated that exposure to sub-lethal doses of ^137^Cs γ ray TBI led to long-term BM suppression and induced a decrease in the peripheral blood cell counts [14,15]. To investigate the SB203580 treatment effects on IR-induced hematopoietic system injury, the numbers of peripheral blood cells were analyzed 80 days after 6 Gy TBI. Mice irradiated with 6 Gy^137^Cs γ rays received injections of SB203580 or vehicle according to the schedules (Figure 1A). The numbers of WBCs, RBCs, HGB, and platelets in the vehicle-treated and SB203580-treated mice exhibited substantial reductions 80 days after 6 Gy TBI compared with the control mice; however, the treatment of the irradiated mice with SB203580 had no effect on the number of WBCs, RBCs, HGB, or platelets compared with the vehicle-treated mice (Figure 1B). These findings suggest that SB203580 treatment 70 days after 6 Gy TBI does not ameliorate TBI-induced hematopoietic system injury.

### 2.2. Effects of SB203580 on BMNC Counts and CFU-GM after TBI

**Figure 2:**
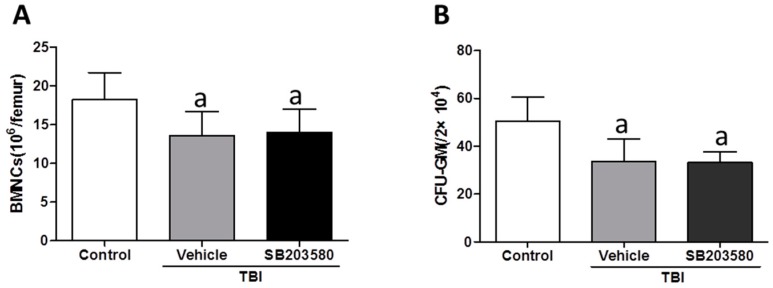
Effects of SB203580 on TBI-induced BMNC counts and CFU-GM. Mice were treated with vehicle or SB203580 70 days after exposure to 6 Gy TBI, as described in the experimental section. (**A**) The number of BMNCs was counted after the mice were euthanized 80 days after 6 Gy TBI; (**B**) The clonogenic function of HPCs in BMNCs was measured via a CFC assay. The data are presented as the means ± SD. *n* = 12 mice/group; ^a^ *p* < 0.05 *vs.* control.

To investigate the effects of SB203580 treatment on TBI-induced long-term BM injury, we initially analyzed the (bone marrow nucleated cell) BMNC counts 80 days after 6 Gy TBI. The number of BMNC in the vehicle-treated and SB203580-treated mice was decreased compared with the control mice. However, the difference between the mice that received SB203580 and vehicle was not statistically significant (Figure 2A). Furthermore, we performed a CFC assay to determine whether SB203580 treatment increased the colony-forming capacity of HPCs from the irradiated mice. Radiation exposure significantly reduced the frequencies of CFU-GM 80 days after 6 Gy TBI (Figure 2B). However, there were no significant differences in the CFU-GM frequencies between the SB203580-treated and vehicle-treated mice. These findings suggest that SB203580 has no effects on the BMNC numbers or colony formation of CFU-GM 70 days after 6 Gy TBI.

### 2.3. Effects of SB203580 on TBI-Induced Long-Term HSC Injury

**Figure 3:**
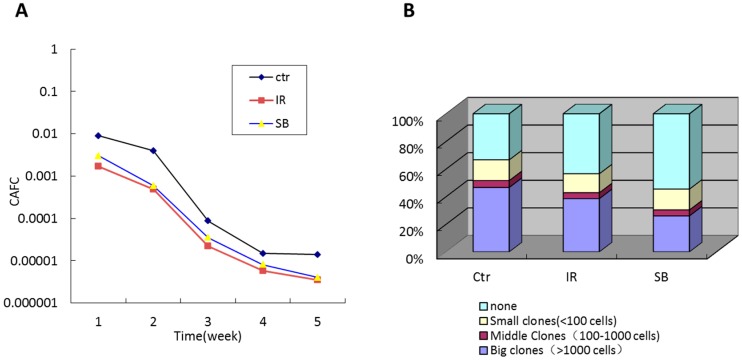
Effects of SB203580 on TBI-induced long-term BM injury. Mice were treated with vehicle or SB203580 70 days after exposure to 6 Gy TBI, as described in the experimental section. BMNCs were collected from the mice 80 days after 6 Gy TBI. (**A**) The clonogenic function of HSCs was measured via a CAFC assay; (**B**) The clonogenic capacity of HSCs in BM was analyzed using a single-cell colony assay.

We subsequently investigated the effects of SB203580 treatment on TBI-induced long-term HSC injury. First, we analyzed the HSC clonogenic function via a cobblestone area-forming cell (CAFC) assay. The 35-day CAFC in the vehicle-treated mice was lower than the control mice. However, the decrease in the HSC clonogenic function was not relieved by SB203580 treatment (Figure 3A). To further validate this result, we used a single-cell colony assay. Similarly, the results demonstrated that SB203580 treatment did not rescue the HSC clonogenic function suppression induced by TBI, which was consistent with the CAFC assay (Figure 3B). These findings suggest that SB203580 does not attenuate TBI-induced long-term HSC suppression.

### 2.4. SB203580 Inhibits TBI-Induced Chronic Oxidative Stress

**Figure 4:**
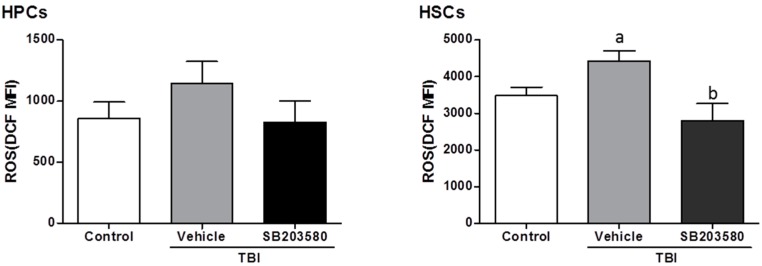
Effects of SB203580 on the TBI-induced increase in ROS in HPCs and HSCs. Mice were treated with vehicle or SB203580 70 days after exposure to 6 Gy TBI, as described in the experimental section. BMNCs were collected from the mice 80 days after 6 Gy TBI, and the levels of intracellular ROS in the HSCs and HPCs were measured. The data are presented as the means ± SD. *n* = 12 mice/group; ^a^ *p* < 0.05 *vs.* control; ^b^ *p* < 0.05 *vs.* TBI + vehicle.

Increasing evidence demonstrates that total-body exposure to radiation in mice induced long-term BM suppression via the induction of chronic oxidative stress and senescence in HSCs [16,17]. Moreover, exposure to a sub-lethal dose of TBI selectively induced high levels of intracellular ROS in HSCs [16,18]. The production of ROS was increased in the HSCs rather than the HPCs from the vehicle-treated mice, even 80 days after 6 Gy TBI, compared with the control mice (Figure 4), which suggests that persistent oxidative stress existed in HSCs 80 days after TBI.

### 2.5. SB203580 Inhibits p38 Expression Augment in HSCs but Not Senescence in HSCs

**Figure 5:**
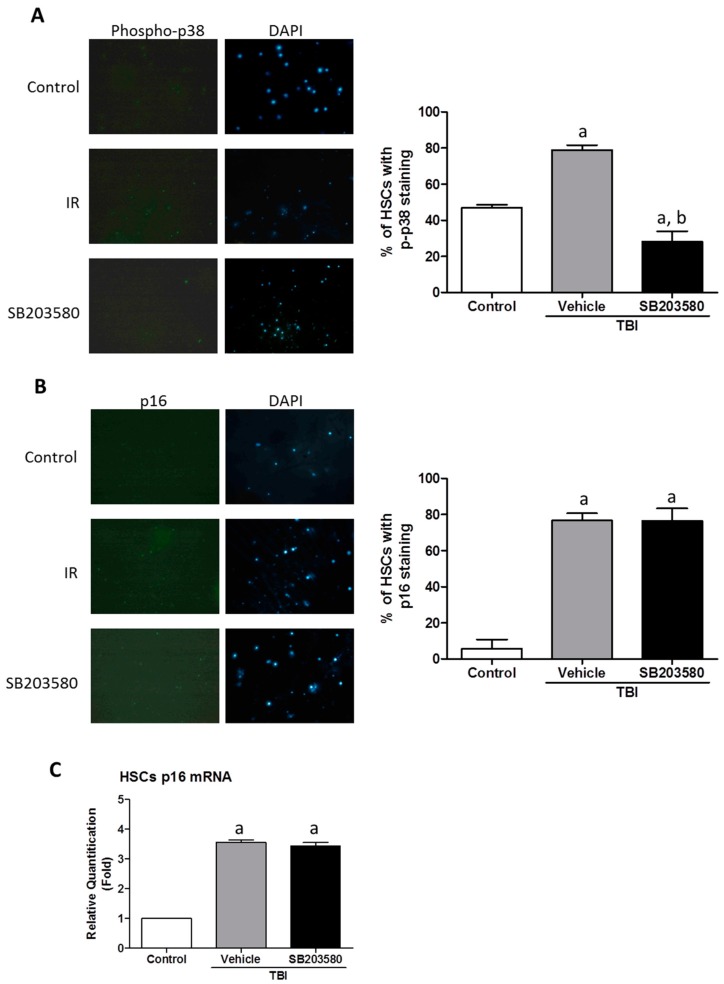
Effects of SB203580 on TBI-induced increase in ROS in HPCs and HSCs. Mice were treated with vehicle or SB203580 70 days after exposure to 6 Gy TBI, as described in the experimental section. BMNCs were collected from the mice 80 days after 6 Gy TBI, and HSCs were isolated from BMNCs by cell sorting. (**A**,**B**) Analysis of the expression of p-p38 and p16 in HSCs. **Left**: representative photomicrographs of p-p38/p16 and DAPI nuclear staining immunostaining in isolated HSCs are shown; **Right**: the percentages of p-p38/p16 positive HSCs are presented as the means ± SD (*n* = 3). Magnification: 10×; (**C**) The levels of p16 mRNA expression in the HSCs were analyzed via RT-PCR. The data are presented as the means ± SEM of the fold changes compared with the control (*n* = 3). ^a^ *p* < 0.05 *vs.* control., ^b^ *p* < 0.05 *vs.* TBI + vehicle.

Research has demonstrated that p38 is crucial for the maintenance of HSC quiescence because it may be activated by ROS [19]. Furthermore, p38 is involved in the mediation of radiation-induced HSC senescence, which is an element attributable to long-term BM injury [9]. Previous studies have demonstrated the vital role of p38 in ROS regulation [16]. The increased levels of ROS led to the induction of HSC senescence, as they expressed a higher level of p16 expression (Figure 5A,B). Treatment with SB203580 inhibited the TBI-induced induction of ROS production in HSCs (Figure 4); however, SB203580 did not reduce the HSC expression of p16 expression. To determine whether SB203580 affects HSC function via the ROS-p38-p16 pathway, we measured the level of p38 phosphorylation (p-p38) in HSCs. TBI induced a substantial increase in p38 activation in HSCs, and SB203580 treatment reduced the p-p38 expression level 80 days after 6 Gy TBI, compared with the vehicle treatment (Figure 5A). These findings suggest that SB203580 affects TBI-induced long-term BM suppression probably by inhibiting TBI-induced chronic oxidative stress and increases in p-p38 expression, which is the ROS-p38 pathway; however, SB203580 did not ameliorate senescence in HSCs.

## 3. Discussion

Bone marrow (BM) suppression is one of the most important side effects of conventional cancer therapy using chemotherapeutic agents and IR. IR-induced hematopoietic system injury is the major cause of death following accidental or intentional exposure to a moderate or lethal dose of total body irradiation (TBI) [16]. Exposure to IR induces not only acute myelosuppression but also long-term BM suppression [14]. In contrast to acute myelosuppression, long-term BM suppression is manifested by a decrease in HSC reserves and a defect in HSC self-renewal; moreover, long-term BM suppression is long-lasting and exhibits little tendency for recovery. Furthermore, an effective treatment against IR-induced long-term BM injury remains to be developed [20,21].

Our previous studies have demonstrated that exposure to high doses of IR caused long-term bone marrow injury, in part, by selectively inducing HSC senescence [10]. The p38 pathway plays an important role in the mediation of HSC senescence induced by IR, and it may be activated by oxidative stress; the inhibition of p38 activation with the small molecule inhibitor SB203580 has been demonstrated to mitigate residual BM injury, in part, via a reduction in HSC senescence [9,22]. Therefore, we initially investigated whether SB203580 affects long-term hematopoietic injury after ionizing radiation-induced HSC senescence in our well-established and characterized mouse model. The current findings demonstrate that TBI decreased peripheral blood cells and BMNC counts, the colony forming capacity of HPCs and HSCs, induced persistent oxidative stress and activated p38 and senescence in HSCs 80 days after 6 Gy TBI. The inhibition of p38 activity with SB203580 had no effective improvement on the TBI-induced reduction of peripheral blood cells and BMNC counts, HPCs, or HSC clonogenic capacity defects. This effect is likely attributed to the SB203580 treatment-induced reduction in ROS production in the HSCs and the expression of p-p38 but not p16, which plays a main role in senescence. Cell senescence is primarily regulated by increased oxidative stress and telomere shortening [23]. HSC senescence induced by IR is related to a remarkable increase in the production of ROS [16], which is persistent and exhibits little tendency for reduction if not timely eliminated. In our study, intracellular ROS in HSCs persisted 70 days after TBI. SB203580 treatment improved the cumulative ROS level; however, this treatment failed to improve the HSC senescence. Therefore, these findings suggest that the short-term administration of SB203580 long-term post-TBI has little effect on improving senescence in HSCs.

## 4. Materials and Methods

### 4.1. Animals and Reagents

Eight- to 10-week-old male C57BL/6J mice were purchased from Vital River (Beijing, China), mice were housed in the Specific Pathogen Free level animal facility at the Institute of Radiation Medicine (IRM), the Chinese Academy of Medical Sciences (CAMS). All experimental procedures were approved by the Institutional Animal Care and Use Committee of the CAMS (Permit Number 1526, 7 April 2015), and written informed consent was obtained from all participants.

SB203580 was purchased from LC Laboratories (Boston, MA, USA). Biotin-conjugated anti-Mouse-CD4 (clone 34 GK1.5), anti-Mouse-CD8 (clone 53-6.7), anti-mouse-CD11b (clone M1/70), anti-mouse-CD45R/B220 (clone RA3-6B2), anti-mouse-Ly6G/Gr-1 (clone RB6-8C5), anti-mouse-Ter-119 (clone Ter-119), anti-mouse-CD117 (c-kit)-APC (clone 2B8), anti-mouse-Ly-6A/EA (Sca-1)-PE/Cy7 (clone D7), and APC-Cy7-conjugated streptavidin were purchased from eBioscience (San Diego, CA, USA). 2,7-dichlorodihydrofluorescein diacetate (DCFDA) was purchased from Sigma-Aldrich (St. Louis, MO, USA). Rabbit anti-p-p38 (ab61241), rabbit anti-p16, and FITC-conjugated goat anti-rabbit antibodies were obtained from Abcam Biotechnology (Cambridge, MA, USA).

### 4.2. Total-Body Irradiation (TBI) and SB203580 Treatment

The mice were randomly divided into three groups: (a) control; (b) TBI + vehicle; and (c) TBI + SB203580. The mice in the TBI + vehicle and TBI + SB203580 groups were exposed to a sub-lethal dose (6 Gy) of ^137^Cs γ rays at a dose rate of 0.79 Gy per minute in an Exposure Instrument (Atomic Energy of Canada Lim, Chalk River, ON, Canana). Seventy days after TBI, the mice in the TBI+SB203580 group were administered SB203580 via intraperitoneal injection (i.p.) at a dose of 15 mg/kg (diluted in DMSO) body weight every other day for 5 injections; the mice in the TBI + vehicle group received the same volume of vehicle at the same frequency. As a control, mice were sham-irradiated and treated with vehicle in a similar manner as described for the SB203580 treatment.

### 4.3. Peripheral Blood Cell and BM Nucleated Cell (BMNC) Counts

Blood was obtained from anesthetized mice via the orbital sinus and was collected in tubes coated with ethylenediaminetetraacetic acid (K_3_EDTA) 1 day after the last intraperitoneal injection. The cell counts included the white blood cells (WBCs), red blood cells (RBCs), hemoglobin (HGB) and platelets, which were counted using a pocH-100i hematology analyzer (Sysmex, Kobe, Japan). BMNCs were isolated and collected as previously described [14]; the cells were counted using a hematology analyzer and expressed as ×10^6^/femur.

### 4.4. Colony-Forming Cell (CFC) Assay

The CFC assays were conducted by culturing BM cells in MethoCult GF M3534 methylcellulose medium (StemCell Technologies, Vancouver, BC, Canada), and the colonies of CFU-granulocyte macrophages (CFU-GMs) with more than 30 cells were counted on day 5 according to the manufacturer’s instructions. The results were expressed as the numbers of CFU-GMs (/2 × 10^4^).

### 4.5. Cobblestone Area-Forming Cell (CAFC) and Single-Cell Colony Assays

The CAFC and single-cell colony assays were used to determine the clonogenic functions of HSCs as previously described [7,10,24].

### 4.6. Analysis of the Levels of Intracellular Reactive Oxygen Species (ROS) via Flow Cytometry

Lineage-negative hematopoietic (Lin*^−^*) cells were isolated as previously described [7]. Approximately 1 × 10^6^ Lin*^−^* cells were stained with anti-Sca-1-PE and anti-c-Kit-APC antibodies. The cells then were labeled with 10 µM 2,7-dichlorodihydrofluorescein diacetate (DCFDA) for 30 min at 37 °C. The mean fluorescence intensity (MFI) of the DCF in HPCs and HSCs was measured by using a flow cytometer (BD Aria FACSII, San Jose, CA, USA) as previously described [25].

### 4.7. Immunofluorescence Staining for p-p38 and p16

Immunofluorescence microscopy was performed according to the method of Wang *et al.* [9]. Briefly, after Lin- cells were stained with anti-Sca-1-PE and anti-c-Kit-APC antibodies, the Lin*^−^*c-kit^+^Sca-1^+^cells were analyzed and sorted using a BD Aria FACSII cell sorter (BD Bioscience, San Jose, CA, USA); the HSCs were cytospun onto slides using a Centrifugal smear machine (Shandon Cytospin 4, Thermo Scientific, Waltham, MA, USA). The cells were subsequently fixed with 4% neutral formalin and permeabilized with 0.2% Triton X-100. After blocking with 5% goat serum, the cells were incubated with anti-p-p38 (1:100) or anti-p16 (1:100) overnight at 4 °C. After being washed with phosphate-buffered saline, the first antibodies were visualized with Alexa Fluor-488-conjugated goat anti-rabbit IgG antibody (1:200). The nuclei were counterstained with DAPI, and the slides were mounted in vectashield as previously described [12].

### 4.8. Quantitative Real-Time PCR

After the Lin*^−^* cells were stained with anti-Sca-1-PE and anti-c-Kit-APC antibodies, Lin*^−^*c-kit^+^Sca-1^+^ cells were collected. Total cellular RNA from approximately 20,000 sorted HSCs was extracted using TRIzol reagent (Life Technologies, Grand Island, NY, USA) following the manufacturer’s instructions. The expression of p16 was determined via real-time PCR according to our previous reports [8,12].

### 4.9. Statistical Analysis

The results were expressed as the mean ± SD, and the data were analyzed by using a one-way analysis of variance (ANOVA). In the event that ANOVA justified *post hoc* comparisons between experimental group means, these were conducted using the Tukey’s multiple paired comparison test. Differences were considered significant at *p* < 0.05. All analyses were performed using GraphPad Prism Software (San Diego, CA, USA).

## 5. Conclusions

Increasing evidence demonstrates that p38 activation plays a critical role in the regulation of the induction of senescence in HSCs [9,19]; however, the present study demonstrates, for the first time, that the ROS-p38 oxidative stress pathway was inhibited 70 days after TBI treatment with SB203580, whereas short-term administration had no significant effect on long-term hematopoietic injury and HSC senescence of mice exposed to a sub-lethal dose of TBI. The effective therapeutic methods for long-term myelosuppression require further investigation.
